# Liver Fat Scores Moderately Reflect Interventional Changes in Liver Fat Content by a Low-Fat Diet but Not by a Low-Carb Diet

**DOI:** 10.3390/nu10020157

**Published:** 2018-01-31

**Authors:** Stefan Kabisch, Sabrina Bäther, Ulrike Dambeck, Margrit Kemper, Christiana Gerbracht, Caroline Honsek, Anna Sachno, Andreas F. H. Pfeiffer

**Affiliations:** 1 Department of Clinical Nutrition, German Institute of Human Nutrition Potsdam-Rehbrücke, Arthur-Scheunert-Allee 114-116, 14558 Nuthetal, Germany; sabrina.baether@charite.de (S.B.); Ulrike.Dambeck@dife.de (U.D.); Margrit.Kemper@dife.de (M.K.); Christiana.Gerbracht@dife.de (C.G.); caroline.honsek@gmail.com (C.H.); anna.sachno@charite.de (A.S.); afhp@dife.de (A.F.H.P.); 2 German Center for Diabetes Research (Deutsches Zentrum für Diabetesforschung e.V.), Ingolstädter Landstraße 1, 85764 Neuherberg, Germany; 3 Department of Geriatrics, Campus Virchow, Charité University Medicine, Augustenburger Platz 1, 13353 Berlin, Germany; 4 Department of Endocrinology, Diabetes and Nutrition, Campus Benjamin Franklin, Charité University Medicine, Hindenburgdamm 30, 12203 Berlin, Germany

**Keywords:** NAFLD, liver fat index, liver fat score, prediction, intervention, diet, low-fat, low-carb

## Abstract

Background: Non-alcoholic fatty liver disease (NAFLD) is a common metabolic disorder all over the world, mainly being associated with a sedentary lifestyle, adiposity, and nutrient imbalance. The increasing prevalence of NAFLD accommodates similar developments for type 2 diabetes and diabetes-related comorbidities and complications. Therefore, early detection of NAFLD is an utmost necessity. Potentially helpful tools for the prediction of NAFLD are liver fat indices. The fatty liver index (FLI) and the NAFLD-liver fat score (NAFLD-LFS) have been recently introduced for this aim. However, both indices have been shown to correlate with liver fat status, but there is neither sufficient data on the longitudinal representation of liver fat change, nor proof of a diet-independent correlation between actual liver fat change and change of index values. While few data sets on low-fat diets have been published recently, low-carb diets have not been yet assessed in this context. Aim: We aim to provide such data from a highly effective short-term intervention to reduce liver fat, comparing a low-fat and a low-carb diet in subjects with prediabetes. Methods: Anthropometric measurements, magnetic resonance (MR)-based intrahepatic lipid (IHL) content, and several serum markers for liver damage have been collected in 140 subjects, completing the diet phase in this trial. Area-under-the-responder-operator-curves (AUROC) calculations as well as cross-sectional and longitudinal Spearman correlations were used. Results: Both FLI and NAFLD-LFS predict liver fat with moderate accuracy at baseline (AUROC 0.775–0.786). These results are supported by correlation analyses. Changes in liver fat, achieved by the dietary intervention, correlate moderately with changes in FLI and NAFLD-LFS in the low-fat diet, but not in the low-carb diet. A correlation analysis between change of actual IHL content and change of single elements of the liver fat indices revealed diet-specific moderate to strong correlations between ΔIHL and changes of measures of obesity, ΔTG, and ΔALT (all low-fat, only) and between ΔIHL and ΔGGT (low-carb, only). With exception for a stronger decrease of triglycerides (TG) levels in the low-carb diet, there is no statistically significant difference in the effect of the diets on anthropometric or serum-based score parameters. Conclusion: While liver fat indices have proved useful in the early detection of NAFLD and may serve as a cost-saving substitute for expensive MR measurements in the cross-sectional evaluation of liver status, their capability to represent interventional changes of liver fat content appears to be diet-specific and lacks accuracy. Liver fat reduction by low-fat diets can be monitored with moderate precision, while low-carb diets require different measuring techniques to demonstrate the same dietary effect.

## 1. Background

Non-alcoholic fatty liver disease (NAFLD) is a common metabolic disorder all over the world, mainly being associated with a sedentary lifestyle, adiposity, and nutrient imbalance. The increasing prevalence of NAFLD accommodates similar developments for type 2 diabetes and diabetes-related comorbidities and complications.

Uncomplicated NAFLD, i.e., hepatosteatosis, itself may progress to the inflammatory state of non-alcoholic steatohepatitis (NASH). Another complication, irreversible hepatic fibrosis, can lead to functional impairments of liver functions, representing hepatic cirrhosis, and finally culminate in primary malignoma of liver tissue.

Therefore, early detection of NAFLD is an utmost necessity. Most imaging techniques provide only fair information on steatosis status and degree. Ultrasound sonography requires experienced examining personnel and sufficient imaging quality to provide qualitative, non-quantitative estimates. Sonoelastography and similar techniques assess liver tissue density, but not necessarily liver fat content. Computed tomography is inferior to magnetic resonance (MR)-based techniques in the assessment of soft tissues and exposes the patient to a huge amount of ionizing radiation. MR tomography also does not provide sufficient sensitivity and specificity for NAFLD detection and graduation.

As one gold standard, MR spectroscopy has been established, but due to high costs and several medical contraindications its use for NAFLD assessment is limited to clinical studies. Liver biopsies, on the other hand, provide a highly specific measurement of intrahepatic lipid (IHL) amount, but heterogeneously distributed liver fat can easily be misinterpreted. In addition to that, tissue samples can only be taken with a significant risk of liver injury, bleeding, bile leakage, and subsequent complications.

Thus, there is need for screening tools to assess and monitor NAFLD with minimal effort, low costs, and no restrictions with relation to cohort structure or individual medical history. Several NAFLD scores have been developed in order to fill this diagnostic gap. The fatty liver index (FLI) comprises markers of obesity (body mass index (BMI) and waist circumference), dyslipidemia (triglycerides (TG)), and liver injury alanine-aminotransferase (GGT). It highly correlates with objective measures of fatty liver disease and predicts most cases of NAFLD [1]. Surprisingly, waist circumference and other measures of visceral adiposity appear to be the main factor in prediction models [2,3]. The NAFLD-liver fat score (NAFLD-LFS) integrates levels of fasting insulin and transaminases as well as presence of Metabolic syndrome. It provides a similarly good NAFLD prediction compared to the FLI [4]. Both FLI and NAFLD-LFS have been designed and validated for Caucasian populations. Recent publications have increased their accuracy by the additional integration of the genetic marker P184L in the PNPL1A1 gene [4,5]. The hepatic steatosis index (HSI) does also highly correlate with liver fat content, measured by imaging, but was validated in an Asian cohort [6]. There is evidence that liver fat indices differ in their precision depending on the ethnicity of the examined individuals [7]. Other indices have been developed in recent years for patients of various ethnicities [8,9,10]. With these scores, early detection of NAFLD is possible, keeping in mind their moderate sensitivity and specificity [11]. In children, prediction by liver fat estimations tends to be more precise, possibly accounting for a stronger linkage between adiposity and metabolic disorder as compared to post-menopausal cohorts [12,13]. As a general limitation, it is noteworthy that all available indices have been validated against ultrasound sonography, which itself does not precisely reflect NAFLD. Other indices themselves require a sonography examination and are therefore prone to examiner-dependent results. Highly sensitive and specific serum markers are costly alternatives with no superiority compared to conventional liver fat indices [14]. Also, sex differences need to be accounted for when using liver fat indices, as some of their components seem to differentially correlate with NAFLD as do the indices themselves [15,16].

Moreover, data on longitudinal NAFLD monitoring is sparse, as only a few studies have investigated whether changes in NAFLD scores mirror changes in actual liver fat content. Investigations on lipid fractions as correlates of therapeutic liver fat reduction were severely underpowered [14]. One publication reported a moderate correlation of longitudinal changes of conventional and modified NAFLD-LFS with liver fat reduction in a lifestyle intervention trial [5]. Another study group demonstrated weak performance of the FLI as a monitoring tool for liver fat reduction in a dietary trial using a low-fat approach [17]. Low-carb diets have not yet been assessed in the same context, leaving the question open as to whether liver fat reduction by such treatments can be monitored by using liver fat indices with the same—moderate—precision. Thus, more data is required to evaluate liver fat scores in their capability to reflect changes of IHL content. As certain lifestyle treatments are not the only promising candidates to reduce liver fat, this proof of functionality for liver fat scores has to be demonstrated independently of means of intervention.

We therefore investigate the statistical relation between changes in two liver fat scores in a human lifestyle intervention trial that features different dietary approaches for metabolic improvement. The ongoing study compares low-fat and low-carb diets in subjects with prediabetes and assesses, amongst other parameters, liver fat content by MR spectroscopy and liver fat scores on the basis of anthropometric measurements and fasted blood samples.

## 2. Methods

Data for this publication are extracted from the ongoing lifestyle intervention trial “Diabetes Nutrition Algorithms in Prediabetes (DiNA-P)” registered at clinicaltrials.gov: NCT 02609243. DiNA-P compares a one-year low-carb or low-fat dietary intervention in subjects with impaired fasting glucose and/or impaired glucose tolerance. The study was conducted in accordance with the Declaration of Helsinki. The ethics committee of the University of Potsdam approved the study protocol in June 2013 (Proposal 10/2013). All subjects provided written informed consent for their participation in the study. Recruitment for this study started in June 2013 and aims for completion in summer 2018. For this present publication, subjects with randomization until November 2015 were selected to ensure completion of the first diet phase and analysis of all required serum parameters.

At baseline, the participants of the DiNA-P study undergo fasting blood sampling, an oral glucose tolerance test, full anthropometry (body weight, height, abdominal circumferences, bio-impedance analysis) and a medical examination. Study volunteers, if suitable and consenting for MR imaging, are subjected to liver MR spectroscopy and abdominal MR imaging according to pre-published specifications [18]. Data from this publication is restricted to subjects with complete data sets on liver fat content and liver fat scores (per-protocol analysis on diet phase completers without respect to dietary in-/compliance). Subjects with overt diabetes mellitus, severe cardiopulmonary, or hepatic, metabolic, psychiatric, infectious, or inflammatory disease are excluded from the entire study. Also, participants with increased ethanol intake (men: above 30 g per day; women: above 20 g per day) are not part of the presented data set.

After screening, inclusion, and baseline assessment with MR, dietary protocols, and pedometry, subjects are 1:1-randomised to two dietary regimes: low-carb or low-fat. Dietary intervention is conducted for 12 months, split up into a first intensive initiation phase of 3 weeks and a 49-week maintenance phase. All assessments from the screening visit are repeated after the first phase and 6 and 12 months after screening.

This publication only reports data from the first diet phase. In this diet phase, both groups are requested to limit their daily energy intake to 1200–1500 kcal. The low-carb diet aims not to exceed a daily intake of 40 g of carbohydrates. The low-fat requires restriction of fat intake below 30% of total energy intake. Despite the long-term goal of increased physical activity, subjects were instructed not to change physical activity before the second diet phase.

FLI and NAFLD-LFS were calculated according to their first publication [1,4].

Statistical analysis entails calculation of area-under-the-responder-operator-curves (AUROC) for the prediction of NAFLD by FLI and NAFLD-LFS at baseline, split for both diet groups. Also, Spearman correlations were used to evaluate the cross-sectional prediction of NAFLD at baseline and the longitudinal monitoring of NAFLD during the first diet phase. Additional correlation analyses were performed to elucidate potential reasons or mechanisms for diet-dependent results.

## 3. Results

One hundred and forty (140) subjects of the ongoing study were selected for the presented data set. Baseline characteristics for both diet groups are presented in Table 1.

**Figure 1:**
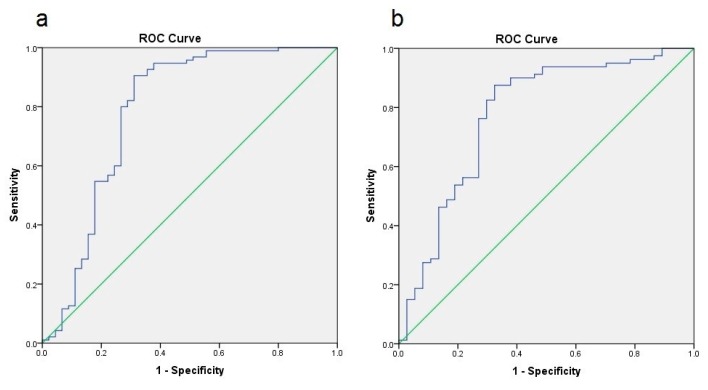
Area-under-the-responder-operator-curves (AUROC) representation of NAFLD prediction by FLI (**a**) and NAFLD-LFS (**b**); both analyses show a significant prediction with AUROC values of 0.786 (FLI) and 0.775 (NAFLD-LFS).

NAFLD prediction by FLI and NAFLD-LFS is comparable to their first publication [1,4], resulting in AUROC values of 0.786 for FLI and 0.775 for NAFLD-LFS in the entire cohort, respectively (Figure 1). AUROC values were comparable for both sub-groups (data not shown).

Accordingly, correlation between liver fat scores and IHL content was strong and highly significant within both diet groups at baseline (Table 2).

In the longitudinal perspective, a moderate, significant positive correlation was present between change of FLI and change of IHL (*r* = 0.499, *p* < 0.001) in the low-fat group. Similarly, a moderate, significantly positive correlation was present between change of NAFLD-LFS and change of IHL (*r* = 0.438, *p* = 0.002) in the low-fat group. In the low-carb group, no such significant correlations could be found (Table 3).

The correlation analysis between change of actual IHL content and change of single elements of the liver fat scores revealed diet-specific moderate to strong correlations between ΔIHL and ΔBMI, Δbody weight, Δwaist circumference, ΔTG, and ΔGGT for the low-fat group, only. A significant, but weak correlation between ΔIHL and ΔALT could be found for the low-carb group, exclusively. Change of IHL and change of fasting insulin correlated significantly but with different magnitude in both groups. Changes of aspartate aminotransferase (AST) and IHL did not correlate significantly (Table 4).

In both diets, actual IHL and almost all of the parameters which were used to calculate FLI and NAFLD-LFS decreased during the diet with statistical significance, but without statistically significant difference between the diets. TG, as the only exception, significantly decreased in both diets, but was significantly stronger in the low-carb diet (data not shown).

## 4. Discussion

Liver fat scores are widely used in metabolic research and clinical practice. Due to low costs, they allow an easy, quick, and relatively accurate prediction of fatty liver disease in patients with a significant risk profile. They do not require imaging procedures and are therefore suitable for patients with claustrophoby, metal implants, or severe obesity. Nevertheless, their measures of sensitivity and specificity leave about 15–20% of all tested subjects with a falsely predicted or falsely excluded diagnosis. Our publication supports the use of these indices for cross-sectional NAFLD prediction before treatment with similar measures of precision.

On the other hand, those indices are also often used to monitor patients during interventions aiming at liver fat reduction despite missing data on the validity of this approach. Our data set implies that liver fat reduction by a low-fat diet is reflected with moderate precision by both scores, while the same metabolic effect on IHL, induced by a low-carb diet, cannot be extrapolated by either FLI or NAFLD-LFS. Similar findings for low-fat diets have been published recently in smaller studies [5,17].

A supplementary correlation analysis indicates that both diets differentially affect single elements of the liver fat scores. A low-fat diet decreases IHL, but also highly correlates to body weight, waist circumference, fasting insulin, triglycerides, and GGT. A low-carb diet leads to IHL reduction, which only correlates with dropping fasting insulin and alanine aminotransferase (ALT) levels.

Several conclusions and hypotheses can be drawn from these findings.

First, under low-carb conditions, IHL reduction appears to be independent from weight loss, rendering BMI or change of BMI a useless parameter for the monitoring of NAFLD and also within certain prediction scores. On the other hand, a strong correlation between weight loss and decreasing liver fat deposits was found for the low-fat group, supporting anthropometric measures as easy surrogate parameters for NAFLD improvement in this particular diet regime.

Second, metabolic improvements of a low-fat diet, particularly a decreasing fasting insulin, seem to be more tightly linked to change in liver fat compared to the amelioration in the low-carb group. Both diets appear to affect liver metabolism and insulin sensitivity via different mechanisms inside and outside of the liver.

Third, reduction of serum TG levels is pronounced in the low-carb group, but does not correlate with IHL improvement. Once again, dietary mechanisms seem to differ between the two regimes, thus modulating the hepatometabolic pattern.

Fourth, change of ALT and IHL reduction correlate under low-carb, but not low-fat conditions. On the other hand, IHL and GGT are linked during a low-fat diet, but not during a low-carb diet. Despite similar baseline values and a similar decrease of both enzyme activities within both diets, the differential correlation with IHL might reflect different subtypes of liver injury that are specifically affected by a certain type of diet.

Some limitations have to be addressed for our analysis. The current data set does not cover all patients from the DiNA-P study, as this trial is still ongoing. Nevertheless, with this paper we provide an analysis that provides a similar statistical power to other papers on this issue.

Also, we are unable to fully clarify the reasons for missing comparability of longitudinal correlations between liver fat scores and MR-based liver fat values between different diets. As a first hint, we demonstrate that both diets differentially affect all components being used to calculate these indices. This highlights that liver fat reduction can be achieved in accompaniment with highly variable metabolic improvements depending on the type of diet. This diet-specific interaction also entails linkage of IHL reduction and total weight loss in low-fat diets, but not low-carb diets.

## 5. Conclusions

Conclusively, with this paper, we replicate the good predictive properties of both FLI and NAFLD-LFS before a dietary intervention in subjects with prediabetes by both AUROC analysis and Spearman correlation. We also demonstrate that actual liver fat reduction is accompanied by moderately correlating liver fat scores in a low-fat, but not a low-carb, diet. This diet-specific finding on correlation magnitude can also be seen for each single component of those liver fat scores.

Liver fat indices need to be used with caution, especially when assessing changes in different interventional settings. Furthermore, more research is needed to elucidate the diet-specific effects of low-carb and low-fat diets on a variety of hepatometabolic disturbances.

## Figures and Tables

**Table 1:** Interventional effect on IHL, liver fat indices and single index parameters.

Parameter	Low-Fat (*n* = 70)	Low-Carb (*n* = 70)	*p*-Value for Comparison Low-Fat versus Low-Carb
Age (years)	60 ± 9	60 ± 11	0.987
Sex (% female)	59%	59%	1.000
Liver fat content (MR-S; %)	11.0 ± 8.5	10.2 ± 7.9	0.589
FLI	68 ± 28	71 ± 26	0.486
NAFLD-LFS	0.56 ± 3.76	−0.07 ± 1.18	0.235
Body weight (kg)	89.0 ± 18.6	91.8 ± 18.0	0.367
BMI (kg/m^2^)	31.1 ± 5.7	32.0 ± 5.6	0.332
Waist circumference (cm)	103.2 ± 15.2	104.1 ± 13.1	0.698
Fasting insulin (pmol/L)	14.2 ± 22.7	9.6 ± 5.5	0.136
Triglycerides (mg/dL)	144 ± 63	143 ± 65	0.968
AST (U/mL)	28 ± 13	27 ± 7	0.503
ALT (U/mL)	30 ± 21	29 ± 13	0.594
GGT (U/mL)	43 ± 63	41 ± 35	0.775

Baseline characteristics; ALT = alanine aminotransferase, AST = aspartate aminotransferase, BMI = body mass index, GGT = gamma-glutamyltransferase, FLI = fatty liver index, MR-S = Magnetic-resonance spectroscopy, NAFLD-LFS = non-alcoholic fatty liver disease-liver fat score; no significant differences between both groups.

**Table 2:** Non-alcoholic fatty liver disease (NAFLD) correlation at baseline.

Correlation between Liver Fat Content (MR-S) and…	Low-Fat (*n* = 70)	Low-Carb (*n* = 70)
FLI	0.550 ***	0.584 ***
NAFLD-LFS	0.532 ***	0.549 ***

Correlation analysis at baseline; FLI = fatty liver index, MR-S = Magnetic-resonance spectroscopy, NAFLD-LFS = non-alcoholic fatty liver disease-liver fat score; *** *p* < 0.001.

**Table 3:** Correlations between change of index values and interventional MR-S data.

Correlation between Change of Liver Fat Content (MR-S) and…	Low-Fat (*n* = 70)	Low-Carb (*n* = 70)
change of FLI	0.499 ***	0.075
change of NAFLD-LFS	0.438 **	0.257

Correlation analysis on interventional changes; FLI = fatty liver index, MR-S = Magnetic-resonance spectroscopy, NAFLD-LFS = non-alcoholic fatty liver disease-liver fat score; ** *p* < 0.01; *** *p* < 0.001.

**Table 4:** Correlations between changes of index parameters and MR-S-based intrahepatic lipid (IHL) data.

Correlation between Change of Liver Fat Content (MR-S) and Change of...	Low-Fat (*n* = 70)	Low-Carb (*n* = 70)
body weight (kg)	0.587 ***	0.082
BMI (kg/m^2^)	0.580 ***	0.072
waist circumference (cm)	0.437 **	0.054
fasting insulin (pmol/L)	0.456 **	0.291 *
triglycerides (mg/dL)	0.371 **	0.215
AST (U/mL)	0.137	0.116
ALT (U/mL)	0.207	0.328 *
GGT (U/mL)	0.420 **	0.247

Correlation analysis on interventional changes; ALT = alanine aminotransferase, AST = aspartate aminotransferase, BMI = body mass index, FLI = fatty liver index, GGT = gamma-glutamyltransferase, MR-S = Magnetic-resonance spectroscopy, NAFLD-LFS = non-alcoholic fatty liver disease-liver fat score; * *p* < 0.05; ** *p* < 0.01; *** *p* < 0.001.

## Abbreviations

ALT	alanine aminotransferase
AST	aspartate aminotransferase
DiNA-P	Diabetes-Nutrition-Algorithms in Prediabetes
FLI	fatty liver index
GGT	gamma-glutamyltransferase
HSI	hepatic steatosis index
NAFLD	non-alcoholic fatty liver disease
NAFLD-LFS	NAFLD-liver fat score
NASH	non-alcoholic steatohepatitis
TG	triglycerides
